# Rethinking academia in a time of climate crisis

**DOI:** 10.7554/eLife.84991

**Published:** 2023-02-07

**Authors:** Anne E Urai, Clare Kelly

**Affiliations:** 1 Leiden University Leiden Netherlands; 2 Trinity College Dublin Dublin Ireland; eLife United Kingdom; eLife United Kingdom

**Keywords:** universities, climate change, activism, research culture, point of view, None

## Abstract

Addressing the climate crisis requires radical and urgent action at all levels of society. Universities are ideally positioned to lead such action but are largely failing to do so. At the same time, many academic scientists find their work impeded by bureaucracy, excessive competitiveness, and a loss of academic freedom. Here, drawing on the framework of “Doughnut Economics,” developed by Kate Raworth, we suggest seven new principles for rethinking the norms of scientific practice. Based on these, we propose a call to action, and encourage academics to take concrete steps towards the creation of a flourishing scientific enterprise that is fit for the challenges of the 21^st^ century.

## Our predicament

The climate and ecological crisis (hereafter, the “climate crisis”) threatens to destabilize many aspects of human civilization – including academic research and education. Our planet’s rapid temperature rise and the unprecedented rate of species loss and ecosystem destruction are a direct result of human activities, predominantly the extraction and consumption of fossil fuels (Thunberg, 2022). Greenhouse gas emissions continue to rise despite decades of political effort (Stoddard et al., 2021). This highlights the powerful role of vested interests, which maintain their legitimacy through engrained norms and the support of societal institutions, including academia (Almond et al., 2022; Popovic et al., 2007). To address the climate crisis, societies must urgently cease supporting and legitimizing policies and practices that harm our biosphere.

Universities have enormous potential to accomplish such transformative change. Academic research expands our knowledge and understanding of the climate crisis, informing policy for mitigation and adaptation. Educating the next generation can set off powerful ripple effects and push society towards the social tipping points required for mass mobilization, action, and system change.

Despite this potential, many academics feel unable to do much about the climate crisis. We may feel our research is unrelated, or that we lack the requisite expertise to engage beyond the realm of personal lifestyle choices. Here, we suggest that an even greater barrier is the fact that the increasingly corporatized, target-driven, and stressful nature of modern academic life far exceeds reasonable human limits. This leaves most of us with no energy to engage with the greatest challenge of our time – tackling the climate crisis. To remove these barriers to action, we need to rethink academia.

To help us in this task, we build on the work of Kate Raworth, whose influential book, *Doughnut Economics*, rethinks economics (Raworth, 2017). Raworth explains how the theories, axioms, and graphical depictions of neoclassical economics have profoundly shaped our world-view. Within the neoclassical frame, humans are rational, selfish, and short-termist – they are separate from, and dominant over, nature.

This justifies a particular kind of economy, where growth is the primary goal, and social goals, such as equality and well-being, are secondary. Nature is a resource; its depletion and destruction an uncosted side-effect. Production and consumption in the free market are paramount, while activity in the household and in the public sphere is undervalued. These foundational assumptions underpin not just economics but have percolated through the political and social norms that govern our societies, right down to our academic institutions. Raworth argues that this outdated economic thinking has led humanity to our current predicament: the twin crises of profound global inequality and climate chaos.

To work our way out of this situation, Raworth suggests that we need to think about economics in a new way (Raworth, 2017). At the heart of the framework is a doughnut, made out of an inner and outer ring: the inner ring is a set of foundations for human wellbeing that we should provide (such as water and food, education and peace; United Nations, 2015); and the outer ring is a set of ecological boundaries for our planet that we must not overshoot (such as pollution, biodiversity loss, and climate change; Steffen et al., 2015).

Raworth’s call to action is that we should not aspire to boundless economic growth, but rather to live well within an ecologically safe and socially just space – the space within the doughnut. This is no easy task; it requires fundamental shifts in our thinking. To facilitate those, Raworth offers seven new principles of economic thought: to change the goal, get savvy with systems, see the big picture, create to regenerate, nurture human nature, design to distribute, and to be agnostic about growth. Raworth’s model has been enormously influential; it has already been used by city planners, industries and businesses worldwide to meet human needs while respecting planetary boundaries (Boffey, 2020).

**Figure 1.**
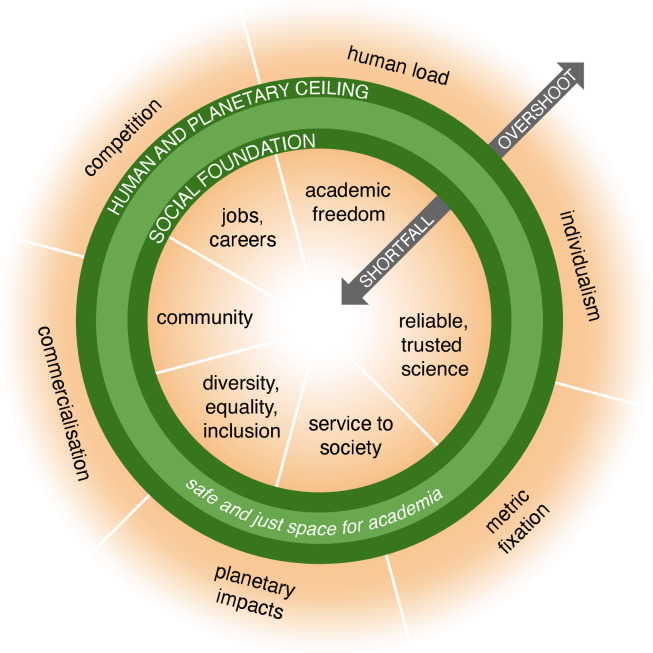
Doughnut academia. Adapting the “doughnut” model of economics to the academic world enables us to visualize the inner social foundations that universities should provide, and the outer human and planetary boundaries that universities need to avoid overshooting. Note that the ordering of elements within the inner and outer rings is random; there is no direct pairing between foundations and ceilings.

Adapted from Raworth, 2017 under a CC-BY-SA license.

**Figure 2.**
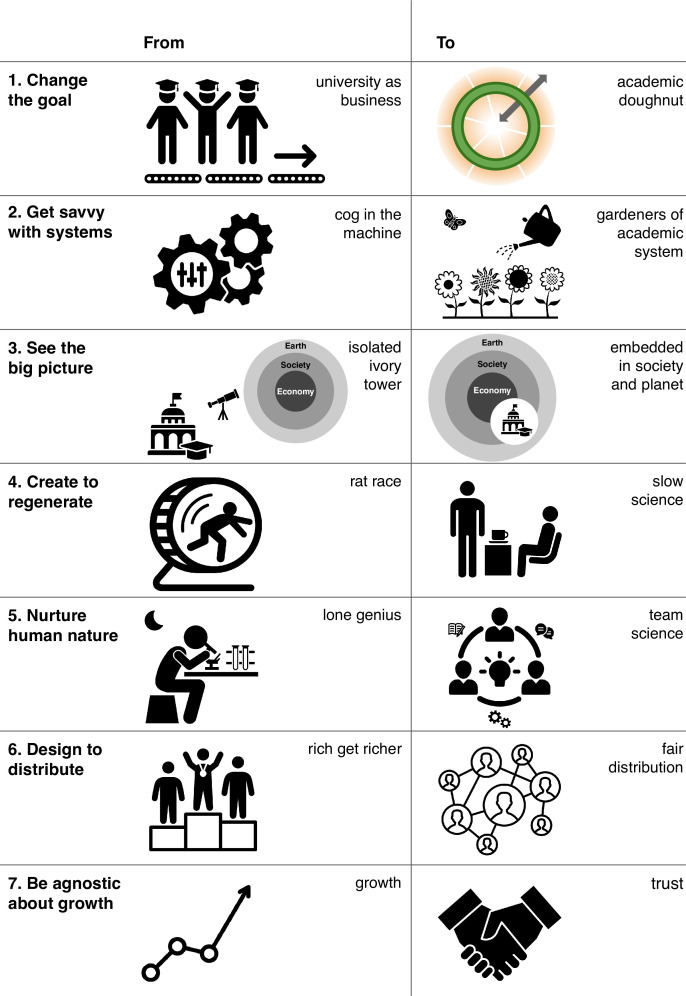
Seven ways to think like a 21st century scientist. *1. Change the goal*: from a business that produces papers and graduated students, towards a university that works towards the inside space of the academic doughnut. *2. Get savvy with systems*: from feeling like a cog in the university machine, towards being gardeners of our academic system. *3. See the big picture*: from academics who look out over the world from their ivory tower, towards scholarship which accepts its own embeddedness in (and dependence on) society and the planet. *4. Create to regenerate*: from a rat race where we tread water, towards “slow scholarship” that values community building, deep thinking and rest crucial for intellectual work. *5. Nurture human nature*: from the lone genius, towards team science. *6. Design to distribute*: from a funding system where the rich get richer, towards a fair distribution of opportunities and resources. *7. Be agnostic about growth*: from a focus on increasing numbers of papers, citations and students, towards rebuilding trust in our own academic communities and with society.

Here, we apply Raworth’s tools for new thinking to our own sphere of academia. In a joint analysis and manifesto, we imagine an “academic doughnut,” bounded by a social foundation and human and planetary ceilings, and describe how academia increasingly under- and overshoots these boundaries (Figure 1). Second, we examine seven dominant, unhelpful ways of thinking, and propose alternatives, which allow us to rethink academia’s future and enable academic action on our planetary crisis (Figure 2). Third, we offer a call to action and suggest concrete steps that each of us can take to move academia into a space where we can thrive in balance (Box 1).

Inspired by Raworth, 2017 under a CC-BY-SA license. Icons from the Noun Project under a CC-BY 3.0 license: ”Graduates” by Adrien Coquet, “University” by Vectors Point, “Telescope“ by Imron Sadewo, “Rat race“ by Luis Prado, “Rest” by ggz, “Lab research“ by Gan Khoon Lay, “Team idea“ by Shashank Singh, “Champion” by !4716068, “Social network” by Josh Sorosky, “Growth“ by Suminah Wulandari, “Agreement“ by DinosoftLab, “Gear“ by Puchongart, “Watering flowers” by Kangriff, “Butterfly” by Bernd Lakenbrink.

Box 1. **From belief to action.**

This paper is intended to start shifting attitudes and foster action. We recognise that a key barrier to any action is time. We cannot wait around for our universities to give us this time – we need to take it back ourselves (Berg and Seeber, 2016). Carve out time to work on the doughnut by putting aside some “bullshit work” (Graeber, 2013; Graeber, 2018). Engaging in work that is consistent with your values and goals will be more rewarding.

Start with 30 minutes this week and try to grow this to something you feel is effective and manageable within your own boundaries. For instance:

- Discuss this paper and the issues it raises (such as work-life balance and activism) with your colleagues or your lab, over lunch, in a journal club or in a book club (see Box 2 for a list of suggested readings).
- Bring an academic doughnut lens to your existing roles when mentoring, reviewing, governing or collaborating.
- Add some slides on the climate and biodiversity crisis at the end of your talks or lectures. Simply indicating that you are worried will resonate with many and open up new conversations.
- Join a sustainability community at your university (e.g., green team or active travel committee). Finding like-minded colleagues is one of the best supports for sustained action.
- Join a local, national or academic climate action group (e.g., Scientists4Future, Scientist Rebellion, Faculty for a Future, ClimateActionNeuPsych, Doctors for XR).

Note that throughout, we will use academia/academics and science/scientists interchangeably. While some of these principles will apply to scientists working outside academic institutions, and many will apply to our colleagues in the arts and humanities, our perspective is primarily informed by our experience as STEM (neuroscience) researchers within universities. We also acknowledge that we speak from positions of privilege, as we are both white, cishet, able-bodied, women academics with tenured positions (Cech, 2022). Our experiences mostly stem from the Irish, Dutch, and American university systems.

## Academia within the doughnut

Adapting the doughnut to academia’s microcosm enables us to visualize a space defined by an inner social foundation (which universities should provide), and outer human and planetary ceilings (which universities need to avoid overshooting).

The social foundation academia should provide:

- *Academic freedom.* Time to think, room for curiosity-driven research.
- *Good jobs and careers.* Work that is valuable and valued, in good, equitable conditions. Secure, satisfying careers with perspective and recognition. Sufficient and equitable resource provision for materials, infrastructure, and scientific support.
- *Community.* Democratic self-governance. Norms and incentives that create healthy, supportive, and collegial communities.
- *Diversity, equality, inclusion.* Freedom of expression and identity. The opportunity to flourish in an academic community without bias and inequality.
- *Service to society.* Societal engagement and input to policy, free from the influence of corporate (e.g., fossil fuel) interests. Responding to society’s needs in our research. Providing high-quality, accessible and affordable higher education options.
- *Reliable, trusted science.* Research that is open, verifiable, and community reviewed. A society that trusts scientists, and science worthy of trust.

The human and planetary boundaries academia should not overshoot:

- *Human load.* Human intellect and creativity, both individual and collective, are academia’s most precious resources. Exceeding this boundary leads to burnout, mental health difficulties, and apathy.
- *Individualism.* The myth of the “lone genius” remains at the heart of the academic picture of success. Overshooting this boundary leads to a devaluation of collaboration and team science and to excessive competitiveness, harassment, and power misuse.
- *Competition.* The gutting of public funding for universities and science has increased competition for scarce resources (grants, publications, promotions, awards), to the detriment of teamwork and collaboration.
- *Metric fixation.* An overshoot of rankings, quantitative metrics, and assessment leads to runaway bureaucracy and perverse incentives to “game the system”. When promotions and hiring processes are yoked to the same goals, the overshoot leads to excessive pressure to publish and to win funding, resulting in irreproducible work, the “rich getting richer”, and academic nepotism.
- *Commercialization.* Public funds should be used to provide common goods, services, and knowledge that benefit society. Excessive commercialization can lead to academic labor being siphoned off by extractive market players (e.g., for-profit publishing, corporate intellectual property, and patents).
- *Planetary impact.* Science and academic research can be a high-resource pursuit. We need to change our own practices to stop overshooting planetary boundaries.

The doughnut’s under- and overshoot will be recognized by many – you may even see these boundaries transgressed in your own academic life. The following sections describe where academia may occupy dangerous operating spaces outside the doughnut and highlight how this modus operandi acts as a primary barrier to meaningful action on the climate and biodiversity crisis. We adapt Raworth’s seven lessons by reviewing hopeful trends and proposing new images to help us move towards a thriving scientific enterprise fit for the 21st century’s challenges.

## Seven ways to think like a 21^st^ century scientist

### Change the goal

Our understanding of the purpose of universities has shifted dramatically over the last few decades (Collini, 2012). The core function of the university is traditionally to preserve, generate and share knowledge and understanding. Since the 1980s, however, universities have increasingly been operated as corporations. This has caused radical changes in what governments, the public, and indeed, academics themselves understand to be the goal of the university (Collini, 2017; Glaser, 2015). Students are viewed as customers who pay (ever increasing) fees for a degree that will serve them in an unpredictable labor market. Academic institutions compete for increasingly scarce public funds, driving a race for reputation, student fees, and grant funding. Curiosity-driven and fundamental research must be justified by appealing to future applications, innovations, and intellectual property (Flexner and Dijkgraaf, 2017). The arts and humanities are devalued, and in some universities, eliminated (Costa, 2019). Pressures to publish (surprising results, in specific outlets, and often) incentivize questionable research practices and even outright fraud. The resulting replication crisis has shaken both scientific communities and the public’s trust in science.

The fixation on metrics manifests as ever-increasing demands for “accountability” – the quantification and justification for every choice made, hour spent, and cent paid. Over coffee, water coolers, and beers worldwide, academics lament how much time is spent “reporting today on the work they said they would do yesterday” (Caitíona Leahy, personal communication). These requirements diminish the time available to do actual work and are deeply immiserating. Even where the underlying goals (e.g., gender equity) have merit, institutional responses often create more work and give only the appearance of having acted, without addressing the underlying problem (Muller, 2018).

Rather than striving for ever-more quantifiable achievements, we must defend what’s unique about the university, an institution for independent scholarship, science, and education for the public good, responsible for “conserving, understanding, extending, and handing on to subsequent generations the intellectual, scientific, and artistic heritage of [hu]mankind” (Collini, 2012). We can do this by realigning our goals towards respecting the academic doughnut’s boundaries. Changing goals, values, and attitudes may be the most difficult of all system changes, but it is ultimately the most important (Meadows, 2008).

### Get savvy with systems

As individuals within the university, we can feel like powerless cogs in the academic machine. After all, who can refuse their Head of Department’s requests for a report on their outputs and impacts, which, if unsatisfactory, will result in the department’s budget being cut? We argue that our ability to resist the forces of metric fixation, competition, and commercialization is impeded by the same barriers that impede climate action: overwork, time poverty, inertia, and the internalization of unhelpful norms (Castiglione et al., 2022; Giurge et al., 2020; Yarkoni, 2018). Many of us are simply too bogged down in busyness and burgeoning bureaucracy to push back on metric fixation in the first place. It is not conspiratorial to suggest, as others have done, that this is not an accident (Glaser, 2015; Graeber, 2013).

To overcome this helplessness, we must see academia not as a research-producing factory but as an organic, diverse, dynamic organization. An organization where, in the right conditions, small seeds can grow into a diverse ecosystem. Just as biology increasingly understands the living world through the lens of dynamical systems, we can observe feedback loops, delays, oscillations, and tipping points in our own professional practice and use these insights to shape the system itself (Meadows, 2008; Nielsen and Qiu, 2022). For example, articulating and questioning the rules of our funding system (and demanding better ways to fund research) can be more worthwhile than writing yet another grant. Taking the time to chat with a student about their interests in climate action, or pushing our university to prioritize sustainability, can be much more impactful than recycling our own coffee cups (Pattee, 2021). It is time to get savvy about the systems we are part of, and to find leverage points for instantiating change within the academy and beyond (Meadows, 1997). This is particularly true for those of us with tenure: career security allows us to stop “playing the game” of harmful academic practices and gives us the moral and practical responsibility to do so.

As scientists, we are uniquely positioned to play our role in the climate movement: we understand complex data (some of us even study human behavior); our expertise is respected and is often influential in driving policy; we work in large organizations from which change can percolate; and we teach the next generation of bright minds. Understanding academia’s systems can help us embrace these social and moral responsibilities to educate and to act (Box 1).

### See the big picture

As academics, we must nurture a self-image that reflects our embeddedness within society and on the planet. This contrasts with the traditional, stereotypical image of academia as an ivory tower, where learned men engage in the noble pursuit of objective and “pure” knowledge. Recent years have witnessed a revision of that image, through a greater appreciation of the reciprocal relationship between science and society, and growing acknowledgement of the fact that academic activities are not value-neutral but are influenced by the values and morals of the culture in which they are embedded. Taking an even wider view, academic activities must respect and protect our planet’s natural systems.

Respecting our societal embedding means recognizing how larger historic and social trends affect academic life, how our biases, privileges, and viewpoints shape the work that we do, and who gets to do that work. It means working for a fair representation of society within communities of students and academics, a goal that we are far from reaching. For instance, US faculty members have a parent with a PhD roughly 25 times more often than the general population, college students predominantly come from wealthy families, and the UK has only 25 black female full professors (Morgan et al., 2022; Aisch et al., 2017; Rollock, 2019). It means acknowledging the legacies of colonialism, and our ongoing complicity in racism, discrimination, and harassment (Ball, 2022). It means working sincerely to confront and eliminate these biases and inequalities by dismantling discriminatory mechanisms (e.g., in admissions, scholarships, hiring, and promotions), and ensuring respect and inclusion. It means attending to whose work we cite and to what and how we teach (Llorens et al., 2021; Begum and Saini, 2019). It means working with and giving back to the communities we research, for instance in co-creation with patient groups, societal partners, and more diverse participant populations (Henrich et al., 2010). In acknowledging our own interconnectedness, we will shed the illusion that research is free from the values and perspectives we bring to it (Gershman, 2021; Kimmerer, 2013; Kuhn, 1962; Saini, 2020).

There are no universities on a dead planet (to borrow a protest slogan): seeing the big picture also means meeting our responsibilities to the planetary systems on which we depend. Our responsibilities can be viewed at two levels: in terms of the carbon and biodiversity footprint of our activities, and in terms of our obligation to research, educate, and activate for structural change (Aron, 2019; Rae et al., 2022). Universities have been surprisingly slow to measure, report, and mitigate the carbon and biodiversity impact of their activities. The changes required have been extensively considered elsewhere, and impact all aspects of the university: from energy use and waste, science lab supplies, to the food sold in university restaurants, and academic travel (Aron et al., 2020; Favaro, 2014; Helmers et al., 2021).

We must not only attend to our carbon footprint but also our “climate shadow”: the total climate impact of our actions, including our impact on others and on society (Pattee, 2021). This means ending activities that support, maintain, and legitimize the fossil fuel industry. Universities must end collaborations with environmentally extractive and other harmful industries, in research, teaching, and funding. Tellingly, research performed in fossil fuel-funded research centers is less favorable towards renewable energy, highlighting how corporate influence can bias scientific outcomes (Almond et al., 2022). Calls for fully “fossil-free research” are beginning to have an impact: universities are starting to reject recruitment and funding from fossil fuel companies (Carrington, 2022; Gilchrist and Kaufman, 2022; Tabuchi, 2022). Universities must also divest their endowments from fossil fuels and from financial institutions that support the fossil fuel industry. More broadly, a shift in our core values is required; the principle of climate justice requires us to center inequality, human rights, and the legacies of colonialism in our approach to the climate and biodiversity crisis (Boyle and Stephens, 2022). As members of the university, we must demand transformative change within our institutions and hold leadership accountable for their commitments and responsibilities (Urai et al., 2022).

### Create to regenerate

To re-evaluate our academic goals, we need time and energy. Freedom from excessive workload and bureaucracy allows us to engage in slow, sustainable, creative work, including curiosity-driven and blue-skies research without obvious monetary or applied value (Berg and Seeber, 2016; Frith, 2020; Haswell, 2017; O’Donovan, 2022). More intellectual breathing space is also crucial to address the breakdown in peer review, mentoring, and other unpaid service that all academics need to do (if the system is to function), but few are willing to take on, since it is scarcely incentivized. Moreover, women (particularly women of color) and members of other underrepresented groups typically carry a disproportionate burden of service work in academia (El-Alayli et al., 2018; Hirshfield and Joseph, 2012).

Happy people who feel valued are the foundation for supportive academic communities that produce creative science. To counter the profound mental health crisis in academia, universities must respect human boundaries and freedom of expression of all identities and must provide a foundation of valued and valuable work (Evans et al., 2018; Wray and Kinman, 2021). Ultimately, this means governments must provide adequate university funding. Unionization and collective action are crucial tools in demanding good, stable careers (Weale, 2022). Those of us with tenure have a particular responsibility to fight against the increasing casualization of academic labor: as many as 70% of academic positions in the US are currently precarious (AAUP, 2018; Burton and Bowman, 2022). While casualized rates in the UK and Europe are lower, striking gender and racial inequalities remain (UCU, 2019; Belleman, 2022). Academic precarity ultimately erodes academic freedom: the ability to express ideas without risk of official interference or professional disadvantage.

Inside the academic doughnut, teaching is central. We educate and invest in the next generation, rather than “bear a load” (Copeland, 2022). To prepare our students to respond to the climate crisis as citizens and professionals, we need to have difficult and honest conversations (Steinberger, 2022b), teach them to think critically and to ask the right questions. Education on the climate crisis must be mainstreamed as part of undergraduate and graduate curricula (Aron, 2019; Molthan-Hill et al., 2019), rather than remaining the focus of a limited number of specialized courses. This requires “teaching the teachers”, providing access to resources and, most important, time for academics to broaden and deepen our own knowledge and understanding. We also need to listen to what young people are telling us about what they want to learn: less about problems, more about solutions and pathways to action (Steinberger, 2022b). Academics across disciplines who take up the challenge of climate crisis education must be rewarded by including such engagement in criteria for promotion, and by offering opportunities to buy-out time to support professional training.

By treating ourselves, our colleagues, and our students as full human beings, rather than just producers of outputs, we can free up space for creativity, collegiality, kindness, and reflection (Berg and Seeber, 2016). This includes working on the climate crisis, which now mostly happens outside people’s day jobs (Gardner et al., 2021). Slowing down and stepping away from the rat race may feel like a strange and radical act, but can ultimately bring great joy into our science, our lives, and the lives of our students.

### Nurture human nature

Our self-image as scientists should nurture human nature, especially our collaborative character. The traditional view remains centered on the lone wolf genius hunched over his desk at night (the image continues to be mostly male and white), as he prepares his high-impact research in solitude. When the image of success is that of the genius or solo research star, it acts as an impediment to the kinds of cooperation and collective action needed to address society’s greatest challenges. Moreover, incentives for individual success enable and even promote power misuse, bullying, and harassment, with ample examples of discrimination and bad behavior throughout the echelons of science (Täuber and Mahmoudi, 2022).

A narrow focus on individual scientific progress ignores the crucial work of building and sustaining communities. Team science, which emphasizes collaboration rather than competition, can better nurture our collegial nature. By teaming up, individuals can focus on sharing their special expertise (from research skills to management, software development, graphical design and outreach) without having to do it all - offering more diverse and fluid career paths outside the role of the principal investigator (Haswell, 2017; Hyman, 2017). At the same time, we must broaden our definition of academic success beyond high-impact papers and grant funding, giving equal value to other domains including mentoring, education, service to discipline, and outreach (Barres, 2013; Nielsen and Nicholas, 2022).

Better ways of crediting and rewarding these collaborative roles in teams is an ongoing effort, which must ultimately be incorporated in hiring, promotion, and funding decisions (Cline et al., 2020). For instance, academia’s values are being re-examined in discussions around recognizing and rewarding scientists in The Netherlands (NWO, 2019). There, national funding bodies and universities have committed to diversifying the dimensions along which individual researchers are assessed, recognizing more diverse career paths that focus on teaching, governance, or outreach. A similar positive step is the Declaration on Research Assessment (DORA), a set of principles that aims to move away from journal-based metrics to assess academics, towards assessing research on its own merits. As academics and reviewers, we can push for our universities and funding agencies to adopt such alternative principles of assessment.

### Design to distribute

Academia is not fair. The distribution of scientific funding, publications in high-impact journals, and the concentration of resources at high-prestige institutions show evidence of the “Matthew effect,” a system where the rich get richer (Bol et al., 2018; Huber et al., 2022). This is dispiriting and wastes academic resources (Gross and Bergstrom, 2019; Roumbanis, 2019; Dresler et al., 2022). It impedes scientific progress by reducing the diversity of people and thought vital to a thriving and resilient scientific community. To ensure sufficient and equitable research support, we need to consider alternative ways of distributing funding, resources, and power. For instance, competitive funding schemes with low success rates (and much time wasted on writing unsuccessful proposals) can be replaced by partial lotteries that free up time and money, without loss of quality (Gross and Bergstrom, 2019; Roumbanis, 2019). Researchers may also be provided with continued rolling grants to provide for basic research needs, pooling together their money to pursue specific projects or redistribute some of their funds to colleagues (Dresler, 2022; Bollen et al., 2014). These alternatives would not only distribute money more fairly, but also free up much-needed time for research, mentoring, activism or even a bit of rest.

The academic doughnut calls for a re-evaluation of how universities use research funds. In recent decades, academics have increasingly served corporate interests through innovations and Intellectual Property generation. Instead, when publicly funded work (such as the development of mRNA vaccines or the PageRank algorithm) is used by private enterprise, the benefits should be fairly and openly shared with the taxpayer to serve the public good. We need to dismantle the publication system that allows academic labor to be siphoned off by publishers with extraordinary profit margins (Resnick and Belluz, 2019). However, with the move towards open access publishing (e.g., Plan S, US regulations) care must be taken to avoid shifting the financial burden onto universities through extortionate Open Access fees. Misuse of community resources by for-profit corporations is widespread (Staller, 2022): Microsoft’s recent release of GitHub Copilot, a programming tool that learns from publicly shared code without respecting its license, met with vocal criticism from the open software community (Gershgorn, 2021). These examples show how applying market principles of commodification, monetization, and private ownership can sour a space that is better served as a community-managed commons (Morrison, 2019; Ostrom, 1990). The success of more disruptive actions such as Sci-Hub (Himmelstein et al., 2018) demonstrates the appetite and capacity for change.

### Be agnostic about growth

The pervasive marketization of universities emphasizes growth: more students getting more degrees, resulting in more quantifiable skills delivered to the labor force; more researchers bringing in more grant money, publishing more papers - that nobody has time to read. Returning to the core question of what universities are actually *for* (Collini, 2012), we need to engage with broader discussions about growthism.

The academic doughnut prioritizes a more organic, growth-agnostic university – where knowledge is preserved, rediscovered and valued, and we nurture what we have. Academic communities are permitted to stop growing so that they can consolidate and bear fruit. The goal should be higher education and a scientific literature that is *better*, rather than *more*: diverse (including code, data, creative works, science communications, or policy documents) and dynamic (freed from the static published journal paper), but slower and smaller in number. These changes will, in turn, open up the breathing space we need to actually engage with that literature (Frith, 2020).

Being growth-agnostic allows us to instead focus on building trust. Questionable research practices (driven ultimately by academia’s incentive structure and the prioritization of incredible findings) have resulted in a crisis of reproducibility, where we can no longer trust the foundations on which we have built our work (Yarkoni, 2018; Vazire, 2019). The open science movement has worked hard to rebuild trust: from meta-analyses, data sharing, and preregistration to team projects and dynamic papers. In parallel, the reproducibility crisis has prompted a renewed focus on theory, which is crucial to improving the usefulness of scientific results (Borsboom, 2013).

The success of open science demonstrates the power of taking back control and designing systems that are distributive by default. Sharing code and data allows others to reproduce scientific work, and levels the playing field for those without access to data acquisition equipment or sufficient funding. Community standards for data help improve scientific quality and reduce waste, by encouraging reuse of data and code formats (Gorgolewski et al., 2016; Teeters et al., 2015). However, open science is not flawless (Mirowski, 2018). For instance, "bro" culture in the open science movement (the problem of “bropen science”) closes the space for diverse voices (Whitaker and Guest, 2020). It is also a challenge to find and fund developers to provide the backbone of open digital infrastructure due to competition with for-profit employers (Bals, 2020). Yet, the grassroots, communal nature of the movement means it has the capacity to be self-reflective and self-correcting. Greater institutional support for open science, in terms of education, funding and secure employment, is crucial for it to be sustainable over the long term.

Ultimately, we need to rebuild society’s trust in science, which has been damaged by increased polarization in public discourse and the rise of populist politicians. The COVID-19 pandemic has laid bare many people’s disenchantment with experts, as exemplified by those who explicitly distance themselves from scientific evidence (Huber, 2022). Trust has been further undermined by the decades-long efforts of the fossil fuel industry to deliberately seed doubt over climate science and to deny their role in the crisis (Supran and Oreskes, 2017). Despite these trends, academics still occupy privileged positions of trust and respect (Clemence and Boyon, 2022). There is a corresponding responsibility to live up to that privileged position by taking time to inform ourselves, and then advocating for change (Gardner et al., 2021; Nielsen and Qiu, 2022). Through collective effort, we can change the academic mantra: from publish, publish, publish, to publish, communicate, engage (Achakulwisut, 2017).

## A call to action

Addressing the climate and biodiversity crisis demands transformative changes in our economies and societies. Academics, both as inhabitants of planet earth and in their professional roles, should take a leading role in this transformation (Gardner et al., 2021). Here, we argue that barriers to the required engagement arise from transgression of the academic doughnut: a safe and just space that provides a social foundation while respecting human and planetary ceilings. To enable academics to engage with humanity’s greatest challenges, and to demand that our institutions lead the way in the required societal transition, we must forge a path towards the safe and just space inside the academic doughnut (Box 2).

Box 2. **Further reading.**

If these ideas resonate with you, we recommend the following books:

- *Doughnut Economics* (Raworth, 2017) examines the axioms of economics and proposes how to live well within planetary boundaries. A clear and ultimately optimistic vision of our future. Videos: TED talk, SR webinar.
- *The Slow Professor* (Berg and Seeber, 2016) proposes a human, regenerative and joyful way of practicing our research, teaching and academic service. A breath of fresh air for the harried scientist.
- *Tyranny of Metrics* (Muller, 2018) describes how “metric fixation” replaces intrinsic with extrinsic rewards, encourages gaming the system, and siphons off time and resources for real work. A readable and broadly useful critique of ever-increasing accounting.
- *What Are Universities For?* (Collini, 2012) returns to the fundamental question of what makes universities special and reviews the historic trends that have led to a breakdown of these values. A foundation for understanding past and current changes in academia.
- *The Climate Crisis* (Aron, 2022) synthesises insights from psychological science on why we're in crisis and what we need to do now to stop global heating. Not just a textbook, but a framework for effective climate action. Adam is a neuroscientist and colleague who recently transitioned his lab from working on cognitive neuroscience to the psychology of the climate crisis and collective action.
- Fabian Dablander curates a reading list that provides a comprehensive overview of the best materials on the climate crisis.

It’s humbling to realize that universities (like many complex systems) show considerable inertia, and that social change does not come easily. Moreover, the academic doughnut cannot be viewed independently of society. Beyond our own institutions, change will ultimately have to come from governments and citizens alike. Many of our proposals are contradictory to the incentive structure of the neoliberal university, defined by dwindling funding and increasing demands for accountability. We also face a conundrum: the changes we want (e.g., sustainability, diversity, open science) can only be explicitly encouraged if we measure and mandate them somehow. Vaguely wishing for culture change is unlikely to achieve much. On the other hand, “not everything that counts can be counted” (Collini, 2012). The academic doughnut cannot be just another requirement; we must change the goal of the university. Given the enormity of this task, do we have a chance of achieving these bold changes?

Here, the past offers hope for the future. Both history and recent experience demonstrates that social movements can and do achieve change – in women’s suffrage, civil rights, and marriage equality (Engler and Engler, 2016). Throughout history, universities have been fertile ground for major social movements, such as the anti-nuclear weapons movement, the anti-war and civil rights movements in the US, and environmental protection movements (Dahlum, 2019; Russell et al., 1955; Brown and Silber, 1979; Racimo et al., 2022). Today, academic activists worldwide make crucial contributions to movements in domains including the climate crisis, health, LGBTQ+ and reproductive rights, social justice, economic inequality, and the hegemony of economic growth, amongst many others (Capstick et al., 2022; Racimo et al., 2022; Dreifus, 2019; Barres, 2006; Tannam, 2018; George, 2020; Holmes, 2021; Hickel et al., 2022).

Within academia, grassroots movements have forced a reckoning with the consequences of several unhelpful practices. While the open science movement was initially met with strong resistance, its practices are becoming increasingly normalized (Nielsen and Qiu, 2022). We can see the fruits of open science activism in concrete initiatives towards new ways of publishing, funding, crediting researchers, and in the overwhelming success of open science initiatives such as data and code sharing (Eisen et al., 2022; Dresler et al., 2022; Zurn et al., 2020). The *#MeToo* movement and its exposure of harassment and abuse offers another example of how activism can bring about system change – though there is still much work to be done. Yet other grassroots movements such as Pride in STEM and #BlackInSTEM (in our own discipline, #BlackInNeuro) are succeeding in calling out inequities and bias, building communities of support, and pushing for true diversity, equality, and inclusion in science.

As is true for any of these examples of success, change starts with collective, sustained, local action (as captured by the motto “think global, act local”). Our academic sphere is where we are and what we can influence, from where change can percolate. While the neoliberal turn has managed to quash some of the rebellious and civic spirit of academics and students, universities are still societal microcosms that can give rise to new ways of thinking, which can trigger systems change. Our individual actions are not *sufficient* to bring about change, but they are *necessary*. Universities will not change without individual, bottom-up efforts from academics (Steinberger, 2022a). As with the climate crisis, we cannot simply wait for someone else to fix the problem.

Crucially, collective action can create an effort multiplier, with long-term effects that can far outstrip any impact we might have through our individual footprints. Talking with colleagues, friends, and family about the climate and biodiversity crisis is one of the most powerful things we can do (Hayhoe, 2021). When we fail to speak up about issues that concern us, we exacerbate a “false social reality” in which the majority appear not to care (Sparkman et al., 2022). Questioning norms can help trigger social tipping points: when just 25% of people support a new social norm, the majority opinion shifts (Centola et al., 2018; Ye et al., 2021). This is why finding your community is so important: solidarity is sustaining, and adding your voice can be a leverage point for more ambitious action. Join a local organization, get involved in politics (as a participant, voter or campaigner), or start a discussion and action group (Box 1). Know that you are not alone: we ourselves have been inspired and guided by the activism and work of our colleagues and students.

If universities do not take a leading role in climate action, we risk being dragged along by larger societal forces. Just five years ago, Greta Thunberg was an unknown Swedish teenager in solitary protest. She has now inspired climate protests in more than 150 countries (Barclay and Resnick, 2019). In this context, there are growing calls for universities to take a more active civic role and to embrace new academic practices, advocacy and more direct climate activism (Aron, 2019; Capstick et al., 2022; Gardner et al., 2021; Racimo et al., 2022). By cultivating, enabling, and rewarding a culture of political advocacy and activism amongst academics, we can be empowered to translate our research beyond the pages of journal publications into real-world impact and action.

Universities are ideally positioned to cultivate the transformative societal, economic, and political change required to address the climate and biodiversity crisis, but can academic communities overcome inertia and barriers to action? A first step towards change is to look at things differently. We have described new ways to view ourselves as researchers, educators, members of the university and of a global scientific community. We hope that our call to action will move us along the path towards the academic doughnut and a thriving biosphere for future generations.

### Citation diversity statement

Recent work in several fields of science has identified a bias in citation practices such that papers from women and other minority scholars are under-cited relative to the number of such papers in the field (Zurn et al., 2020). Here, we sought to proactively choose diverse references. Our references contain 36% woman/woman, 12% man/woman, 10% woman/man and 42% man/man (reflecting the first and last author, where a single author was counted as both first and last).

## Data Availability

No data were generated for this article.

## References

[bib1] AAUP (2018). Data Snapshot: Contingent Faculty in US Higher Ed. https://www.aaup.org/news/data-snapshot-contingent-faculty-us-higher-ed.

[bib2] Achakulwisut P (2017). Why are scientists so averse to public engagement?. Scientific American.

[bib3] Aisch G, Buchanan L, Cox A, Quealy K (2017). Some colleges have more students from the top 1 percent than the bottom 60. Find yours. New York Times.

[bib4] Almond D, Du X, Papp A (2022). Favourability towards natural gas relates to funding source of university energy centres. Nature Climate Change.

[bib5] Aron AR (2019). The climate crisis needs attention from cognitive scientists. Trends in Cognitive Sciences.

[bib6] Aron AR, Ivry RB, Jeffery KJ, Poldrack RA, Schmidt R, Summerfield C, Urai AE (2020). How can neuroscientists respond to the climate emergency?. Neuron.

[bib7] Aron AR (2022). The Climate Crisis: Science, Impacts, Policy, Psychology, Justice, Social Movements.

[bib8] Ball P (2022). UK institutions confront the shadows of imperialism. Nature.

[bib9] Bals F (2020). TANSTAAFL! The tragedy of the commons meets open source software. Synopsys.

[bib10] Barclay E, Resnick B (2019). How big was the global climate strike? 4 million people, activists estimate. Vox.

[bib11] Barres BA (2006). Does gender matter?. Nature.

[bib12] Barres BA (2013). How to pick a graduate advisor. Neuron.

[bib13] Begum N, Saini R (2019). Decolonising the curriculum. Political Studies Review.

[bib14] Belleman B (2022). Temporary university contracts rise again, despite protests. U-Today.

[bib15] Berg M, Seeber BK (2016). The Slow Professor: Challenging the Culture of Speed in the Academy.

[bib16] Boffey D (2020). Amsterdam to embrace “doughnut” model to mend post-coronavirus economy. The Guardian.

[bib17] Bol T, de Vaan M, van de Rijt A (2018). The matthew effect in science funding. PNAS.

[bib18] Bollen J, Crandall D, Junk D, Ding Y, Börner K (2014). From funding agencies to scientific agency. EMBO Reports.

[bib19] Borsboom D (2013). Theoretical Amnesia. Open Science Collaboration.

[bib20] Boyle AD, Stephens JC (2022). Higher education needs a new mission. How about climate justice?. Boston Globe.

[bib21] Brown BA, Silber G (1979). The War at Home. https://www.thewarathome.tv/.

[bib22] Burton S, Bowman B (2022). The academic precariat: Understanding life and labour in the neoliberal academy. British Journal of Sociology of Education.

[bib23] Capstick S, Thierry A, Cox E, Berglund O, Westlake S, Steinberger JK (2022). Civil disobedience by scientists helps press for urgent climate action. Nature Climate Change.

[bib24] Carrington D (2022). Fossil fuel recruiters banned from three more UK universities. The Guardian.

[bib25] Castiglione A, Brick C, Holden S, Miles-Urdan E, Aron AR (2022). Discovering the psychological building blocks underlying climate action: A longitudinal study of real-world activism. Royal Society Open Science.

[bib26] Cech EA (2022). The intersectional privilege of white able-bodied heterosexual men in STEM. Science Advances.

[bib27] Centola D, Becker J, Brackbill D, Baronchelli A (2018). Experimental evidence for tipping points in social convention. Science.

[bib28] Clemence M, Boyon N (2022). Doctors and scientists are seen as the world’s most trustworthy professions. Ipsos.

[bib29] Cline H, Coolen L, de Vries S, Hyman S, Segal R, Steward O (2020). Recognizing team science contributions in academic hiring, promotion, and tenure. Journal of Neuroscience.

[bib30] Collini S (2012). What Are Universities For?.

[bib31] Collini S (2017). Speaking of Universities.

[bib32] Copeland P (2022). Stop describing academic teaching as a “load.”. Nature.

[bib33] Costa RC (2019). The place of the humanities in today’s knowledge society. Palgrave Communications.

[bib34] Dahlum S (2019). Students in the streets: education and nonviolent protest. Comparative Political Studies.

[bib35] Dreifus C (2019). Gregg gonsalves blends activism and science. The New York Times.

[bib36] Dresler M (2022). FENS-kavli network of excellence: postponed, non-competitive peer review for research funding. European Journal of Neuroscience.

[bib37] Dresler M, Buddeberg E, Endesfelder U, Haaker J, Hof C, Kretschmer R, Pflüger D, Schmidt F (2022). Why many funding schemes harm rather than support research. Nature Human Behaviour.

[bib38] Eisen MB, Akhmanova A, Behrens TE, Diedrichsen J, Harper DM, Iordanova MD, Weigel D, Zaidi M (2022). Peer review without gatekeeping. eLife.

[bib39] El-Alayli A, Hansen-Brown AA, Ceynar M (2018). Dancing backwards in high heels: Female professors experience more work demands and special favor requests, particularly from academically entitled students. Sex Roles.

[bib40] Engler M, Engler P (2016). This Is an Uprising: How Nonviolent Revolt Is Shaping the Twenty-First Century.

[bib41] Evans TM, Bira L, Gastelum JB, Weiss LT, Vanderford NL (2018). Evidence for a mental health crisis in graduate education. Nature Biotechnology.

[bib42] Favaro B (2014). A carbon code of conduct for science. Science.

[bib43] Flexner A, Dijkgraaf R (2017). The Usefulness of Useless Knowledge.

[bib44] Frith U (2020). Fast lane to slow science. Trends in Cognitive Sciences.

[bib45] Gardner CJ, Thierry A, Rowlandson W, Steinberger JK (2021). From publications to public actions: the role of universities in facilitating academic advocacy and activism in the climate and ecological emergency. Frontiers in Sustainability.

[bib46] George N (2020). Angela Davis still believes america can change. New York Times.

[bib47] Gershgorn D (2021). Can AI learn from any public code online?. The Verge.

[bib48] Gershman SJ (2021). Just looking: The innocent eye in neuroscience. Neuron.

[bib49] Gilchrist C, Kaufman C (2022). Princeton activists just won a historic victory for climate research. The Nation.

[bib50] Giurge LM, Whillans AV, West C (2020). Why time poverty matters for individuals, organisations and nations. Nature Human Behaviour.

[bib51] Glaser E (2015). Bureaucracy: Why won’t scholars break their paper chains?. Times Higher Education.

[bib52] Gorgolewski KJ, Auer T, Calhoun VD, Craddock RC, Das S, Duff EP, Flandin G, Ghosh SS, Glatard T, Halchenko YO, Handwerker DA, Hanke M, Keator D, Li X, Michael Z, Maumet C, Nichols BN, Nichols TE, Pellman J, Poline JB, Rokem A, Schaefer G, Sochat V, Triplett W, Turner JA, Varoquaux G, Poldrack RA (2016). The brain imaging data structure, a format for organizing and describing outputs of neuroimaging experiments. Scientific Data.

[bib53] Graeber D (2013). On the phenomenon of bullshit jobs. https://www.atlasofplaces.com/essays/on-the-phenomenon-of-bullshit-jobs/.

[bib54] Graeber D (2018). Are you in a BS job? In academe you're hardly alone. https://davidgraeber.org/articles/are-you-in-a-bs-job-in-academe-youre-hardly-alone/.

[bib55] Gross K, Bergstrom CT (2019). Contest models highlight inherent inefficiencies of scientific funding competitions. PLOS Biology.

[bib56] Haswell ES (2017). The sustainable professor. eLife.

[bib57] Hayhoe K (2021). Saving Us: A Climate Scientist’s Case for Hope and Healing in A Divided World.

[bib58] Helmers E, Chang CC, Dauwels J (2021). Carbon footprinting of universities worldwide: part I—objective comparison by standardized metrics. Environmental Sciences Europe.

[bib59] Henrich J, Heine SJ, Norenzayan A (2010). The weirdest people in the world?. Behavioral and Brain Sciences.

[bib60] Hickel J, Kallis G, Jackson T, O’Neill DW, Schor JB, Steinberger JK, Victor PA, Ürge-Vorsatz D (2022). Degrowth can work - here’s how science can help. Nature.

[bib61] Himmelstein DS, Romero AR, Levernier JG, Munro TA, McLaughlin SR, Greshake Tzovaras B, Greene CS (2018). Sci-hub provides access to nearly all scholarly literature. eLife.

[bib62] Hirshfield LE, Joseph TD (2012). 'We need a woman, we need a black woman’: Gender, race, and identity taxation in the academy. Gender and Education.

[bib63] Holmes M (2021). David Graeber’s real contribution to Occupy Wall Street wasn’t a phrase – It was a process. https://novaramedia.com/2021/09/04/david-graebers-real-contribution-to-occupy-wall-street-wasnt-a-phrase-it-was-a-process/.

[bib64] Huber MT (2022). Climate Change as Class War: Building Socialism on a Warming Planet.

[bib65] Huber J, Inoua S, Kerschbamer R, König-Kersting C, Palan S, Smith VL (2022). Nobel and novice: Author prominence affects peer review. PNAS.

[bib66] Hyman S (2017). Biology needs more staff scientists. Nature.

[bib67] Kimmerer R (2013). Braiding Sweetgrass: Indigenous Wisdom, Scientific Knowledge and the Teachings of Plants.

[bib68] Kuhn TS (1962). The Structure of Scientific Revolutions.

[bib69] Llorens A, Tzovara A, Bellier L, Bhaya-Grossman I, Bidet-Caulet A, Chang WK, Cross ZR, Dominguez-Faus R, Flinker A, Fonken Y, Gorenstein MA, Holdgraf C, Hoy CW, Ivanova MV, Jimenez RT, Jun S, Kam JWY, Kidd C, Marcelle E, Marciano D, Martin S, Myers NE, Ojala K, Perry A, Pinheiro-Chagas P, Riès SK, Saez I, Skelin I, Slama K, Staveland B, Bassett DS, Buffalo EA, Fairhall AL, Kopell NJ, Kray LJ, Lin JJ, Nobre AC, Riley D, Solbakk AK, Wallis JD, Wang XJ, Yuval-Greenberg S, Kastner S, Knight RT, Dronkers NF (2021). Gender bias in academia: a lifetime problem that needs solutions. Neuron.

[bib70] Meadows DH (1997). Leverage points: Places to intervene in a system. https://donellameadows.org/archives/leverage-points-places-to-intervene-in-a-system/.

[bib71] Meadows DH (2008). Thinking in Systems: A Primer.

[bib72] Mirowski P (2018). The future(s) of open science. Social Studies of Science.

[bib73] Molthan-Hill P, Worsfold N, Nagy GJ, Leal Filho W, Mifsud M (2019). Climate change education for universities: A conceptual framework from an international study. Journal of Cleaner Production.

[bib74] Morgan AC, LaBerge N, Larremore DB, Galesic M, Brand JE, Clauset A (2022). Socioeconomic roots of academic faculty. Nature Human Behaviour.

[bib75] Morrison DH (2019). Open access versus the commons, or steps towards developing commons to sustain open access. Sustaining the Knowledge Commons.

[bib76] Muller JZ (2018). The Tyranny of Metrics.

[bib77] Nielsen KS, Nicholas KA (2022). Taking research from idea to impact. http://sustainabilitycommunity.springernature.com/posts/taking-research-from-idea-to-impact.

[bib78] Nielsen M, Qiu K (2022). A Vision of Metascience. https://scienceplusplus.org/metascience/index.html.

[bib79] NWO (2019). Room for Everyone’s Talent. VSNU, NFU, KNAW, NWO and Zon MW.

[bib80] O’Donovan B (2022). Companies report success with trial of four-day week. RTE.

[bib81] Ostrom E (1990). Governing the Commons: The Evolution of Institutions for Collective Action.

[bib82] Pattee E (2021). Forget your carbon footprint. Let’s talk about your climate shadow. MIC.

[bib83] Popovic S, Djinovic S, Milivojevic A, Merriman H, Marovic I (2007). CANVAS Core Curriculum: A Guide to Effective Nonviolent Struggle.

[bib84] Racimo F, Valentini E, Rijo De León G, Santos TL, Norberg A, Atmore LM, Murray M, Hakala SM, Olsen FA, Gardner CJ, Halder JB (2022). The biospheric emergency calls for scientists to change tactics. eLife.

[bib85] Rae CL, Farley M, Jeffery KJ, Urai AE (2022). Climate crisis and ecological emergency: why they concern (neuro)scientists, and what we can do. Brain and Neuroscience Advances.

[bib86] Raworth K (2017). Doughnut Economics: Seven Ways to Think Like a 21st-Century Economist.

[bib87] Resnick B, Belluz J (2019). The war to free science. Vox.

[bib88] Rollock N (2019). Staying power: The career experiences and strategies of UK black female professors. UCU.

[bib89] Roumbanis L (2019). Peer review or lottery? A critical analysis of two different forms of decision-making mechanisms for allocation of research grants. Science, Technology, & Human Values.

[bib90] Russell B, Einstein A, Born M, Bridgman P, Infeld L, Joliot-Curie F, Muller H (1955). The Russell-Einstein Manifesto. Pugwash Conferences on Science and World Affairs.

[bib91] Saini A (2020). Want to do better science? Admit you’re not objective. Nature.

[bib92] Sparkman G, Geiger N, Weber EU (2022). Americans experience a false social reality by underestimating popular climate policy support by nearly half. Nature Communications.

[bib93] Staller KM (2022). Beware the kudzu: Corporate creep, university consumers, and epistemic injustice. Qualitative Social Work.

[bib94] Steffen W, Richardson K, Rockström J, Cornell SE, Fetzer I, Bennett EM, Biggs R, Carpenter SR, de Vries W, de Wit CA, Folke C, Gerten D, Heinke J, Mace GM, Persson LM, Ramanathan V, Reyers B, Sörlin S (2015). Planetary boundaries: Guiding human development on a changing planet. Science.

[bib95] Steinberger JK (2022a). Individuals and social pressure: How to change the world. Brave New Europe.

[bib96] Steinberger JK (2022b). The kids are not OK. Yale Climate Connections.

[bib97] Stoddard I, Anderson K, Capstick S, Carton W, Depledge J, Facer K, Gough C, Hache F, Hoolohan C, Hultman M, Hällström N, Kartha S, Klinsky S, Kuchler M, Lövbrand E, Nasiritousi N, Newell P, Peters GP, Sokona Y, Stirling A, Stilwell M, Spash CL, Williams M (2021). Three decades of climate mitigation: Why haven’t we bent the global emissions curve?. Annual Review of Environment and Resources.

[bib98] Supran G, Oreskes N (2017). Assessing ExxonMobil’s climate change communications (1977–2014). Environmental Research Letters.

[bib99] Tabuchi H (2022). Kicking oil companies out of school. New York Times.

[bib100] Tannam E (2018). 1,200 members of Irish science community sign petition for Yes vote. Silicon Republic.

[bib101] Täuber S, Mahmoudi M (2022). How bullying becomes a career tool. Nature Human Behaviour.

[bib102] Teeters JL, Godfrey K, Young R, Dang C, Friedsam C, Wark B, Asari H, Peron S, Li N, Peyrache A, Denisov G, Siegle JH, Olsen SR, Martin C, Chun M, Tripathy S, Blanche TJ, Harris K, Buzsáki G, Koch C, Meister M, Svoboda K, Sommer FT (2015). Neurodata without borders: creating a common data format for neurophysiology. Neuron.

[bib103] Thunberg G (2022). The Climate Book.

[bib104] UCU (2019). Precarious work in higher education: Insecure contracts and how they have changed over time. UCU.

[bib105] United Nations (2015). Transforming our World: The 2030 Agenda for Sustainable Development. https://sdgs.un.org/publications/transforming-our-world-2030-agenda-sustainable-development-17981.

[bib106] Urai AE, Fossen T, Burtscher L (2022). Why scientists should take the lead in the climate crisis. Mare Online.

[bib107] Vazire S (2019). Do we want to be credible or incredible?. APS Observer.

[bib108] Weale S (2022). Thousands of UK university staff strike over pension cuts. The Guardian.

[bib109] Whitaker K, Guest O (2020). Bropenscience is broken science. British Psychological Society.

[bib110] Wray S, Kinman G (2021). Supporting staff wellbeing in higher education. https://www.educationsupport.org.uk/media/x4jdvxpl/es-supporting-staff-wellbeing-in-he-report.pdf.

[bib111] Yarkoni AT (2018). No, it’s not The Incentives—It’s you. https://www.talyarkoni.org/blog/2018/10/02/no-its-not-the-incentives-its-you/.

[bib112] Ye M, Zino L, Mlakar Ž, Bolderdijk JW, Risselada H, Fennis BM, Cao M (2021). Collective patterns of social diffusion are shaped by individual inertia and trend-seeking. Nature Communications.

[bib113] Zurn P, Bassett DS, Rust NC (2020). The Citation Diversity Statement: A practice of transparency, A way of life. Trends in Cognitive Sciences.

